# Neurotoxic Effect of Myricitrin in Copper-Induced Oxidative Stress Is Mediated by Increased Intracellular Ca^2+^ Levels and ROS/p53/p38 Axis

**DOI:** 10.3390/antiox14010046

**Published:** 2025-01-03

**Authors:** Ignacija Vlašić, Antonio Krstačić-Galić, Anđela Horvat, Nada Oršolić, Anja Sadžak, Lucija Mandić, Suzana Šegota, Maja Jazvinšćak Jembrek

**Affiliations:** 1 Division of Molecular Medicine, Ruđer Bošković Institute, 10000 Zagreb, Croatia; ignacija.vlasic@irb.hr (I.V.); andjela.horvat@irb.hr (A.H.); 2 Department of Biology, Faculty of Science, University of Zagreb, 10000 Zagreb, Croatianada.orsolic@biol.pmf.hr (N.O.); 3 Division of Physical Chemistry, Ruđer Bošković Institute, 10000 Zagreb, Croatia; anja.sadzak@irb.hr (A.S.); lucija.mandic@irb.hr (L.M.); 4 Department of Psychology, Catholic University of Croatia, 10000 Zagreb, Croatia

**Keywords:** flavonol, metal-induced neurotoxicity, pro-oxidative activity, apoptosis, atomic force microscopy

## Abstract

Although commonly appreciated for their anti-oxidative and neuroprotective properties, flavonoids can also exhibit pro-oxidative activity, potentially reducing cell survival, particularly in the presence of metal ions. Disrupted copper homeostasis is a known contributor to neuronal dysfunction through oxidative stress induction. This study investigated the effects of myricitrin (1–20 μg/mL) on copper-induced toxicity (0.5 mM CuSO_4_) in the neuroblastoma SH-SY5Y cell line. At non-toxic concentrations, myricitrin exacerbated copper’s toxic effects. The myricitrin-induced decrease in survival was accompanied with increased reactive oxygen species (ROS) production, reduced superoxide dismutase activity, and a lower GSH/GSSG ratio. In combination with copper, myricitrin also activated caspase-3/7, promoted nuclear chromatin changes, and compromised membrane integrity. At the protein level, myricitrin upregulated p53 and PUMA expression. The toxic effects of myricitrin were alleviated by the p38 inhibitor SB203580, the intracellular calcium chelator BAPTA-AM, and the NMDA receptor blocker MK-801, highlighting the significant role of the ROS/p53/p38 axis in cell death and the critical involvement of calcium ions in apoptosis induction. The atomic force microscopy was used to assess the surface morphology and nanomechanical properties of SH-SY5Y cells, revealing changes following myricitrin treatment. This research highlights the toxic potential of myricitrin and emphasizes the need for caution when considering flavonoid supplementation in conditions with elevated copper levels.

## 1. Introduction

Copper ions, serving as catalytic cofactors or structural components of various enzymes, are essential for normal brain function, influencing various cellular processes, such as the maintenance of redox balance, metabolic activity, synaptic transmission, neurotransmitter synthesis, myelination, intracellular signalling, and gene expression [1,2,3,4].

At the cellular level, copper uptake, transport, storage, and export are tightly regulated by chaperones, copper transporters, and copper coordinators like thiols, ensuring availability for copper-containing enzymes while preventing the toxic accumulation of free ions [5,6]. When copper levels become elevated, as seen in ageing or certain environmental conditions, unbound copper may cause cellular damage, either directly, through non-specific interactions with amino acid residues and DNA nucleotides, or indirectly by catalyzing the production of reactive oxygen species (ROS) in redox cycling reactions [6]. Excessive ROS production leads to oxidative stress, a condition where ROS generation overwhelms the cell’s detoxification capacity, surpassing the capabilities of enzymatic and non-enzymatic antioxidant defence systems [6,7,8].

Copper dyshomeostasis has been implicated in various central nervous system (CNS) diseases [9]. Disturbed levels of copper and imbalances in other metals, particularly iron and zinc, have been observed in neurodegenerative conditions, including Parkinson’s disease and Alzheimer’s disease. Studies suggest that copper dysregulation in vulnerable brain areas plays a critical role in the etiopathogenesis of these diseases, mainly by promoting oxidative stress that leads to neuronal dysfunction [10,11,12]. Ultimately, an imbalance in redox homeostasis and elevated ROS production can alter signal transduction across various pathways, influencing cell survival and death. Mitogen-activated protein kinase (MAPK) pathways, especially the p38 kinase pathway, play a significant role in determining neuronal cell fate under oxidative stress and in various neurodegenerative disorders [13,14]. All isoforms of p38 kinase are activated by upstream kinases, with mitogen-activated protein kinase kinases 3 and 6 (MKK3/6) being highly specific for p38. These kinases are phosphorylated (activated) by a variety of other kinases in response to diverse extracellular stimuli, including ROS [14,15]. The substrates of p38 kinase include other kinases, transcription factors, and cytoskeletal proteins, all of which may be involved in neurodegenerative processes [14]. Moreover, p38 MAPK activation can lead to further ROS production, creating a vicious cycle that amplifies the neuronal response to oxidative stress [14]. Increased intracellular calcium levels can also transduce the signal to p38 cascade, contributing to neuronal degeneration [13,16]. Moreover, elevated calcium levels can further increase ROS production via several mechanisms, such as the induction of mitochondrial dysfunction and the activation of NADPH oxidases and various kinases, including the p38 kinase. The activation of these kinases in turn can stimulate ROS-generating enzymes and exacerbate ROS production [17,18]. Despite extensive research, effective therapies for oxidative stress-related neurodegenerative conditions are still lacking. Current therapies mainly focus on alleviating symptoms rather than addressing the underlying causes or slowing the disease’s progression, leading to significant medical, economic, and social burdens [19]. Given the complexity of mechanisms involved in the development and progression of neurodegenerative diseases, especially those related to oxidative stress, redox imbalance, and redox-sensitive pathways, there is a growing interest in multitarget therapeutic compounds. Such compounds could simultaneously address various mechanisms underlying neuronal dysfunction, potentially offering more comprehensive benefits [20,21,22]. Flavonoids, known for their pleiotropic activities, have emerged as promising neuroprotective agents [23,24]. Flavonols, a class of flavonoids commonly found in the human diet, have demonstrated protective effects in various in vitro and in vivo models involving elevated ROS [25,26,27]. These compounds act as radical scavengers [28,29], metal chelators [30], and modulators of antioxidant defence [31,32], while also regulating key signalling pathways [33,34]. However, flavonols can show either protective (anti-oxidative) or detrimental (pro-oxidative) effects in the presence of metal ions. The outcome depends on the concentrations of both flavonols and metal ions [35,36], the number and positions of hydroxyl groups, metal chelation capacity [37,38], and the cellular oxidative stress context, particularly GSH levels [39,40]. Therefore, understanding the cellular and molecular mechanisms that determine whether flavonols and other polyphenolic compounds will act as antioxidants or prooxidants is essential for developing safe and effective therapies for neurological disorders [41].

Myricitrin (C_21_H_20_O_12_, myricetin-3-O-α-rhamnoside) (Figure S1), a flavonol found in *Myrica rubra* and other dietary plants, is used as a flavour modifier in food and beverages [42]. Although much less studied than other flavonols, myricitrin has shown anti-oxidative, anti-inflammatory and neuroprotective potential in in vitro and in vivo settings. Thus, in PC12 cells, it mitigated ROS production induced by 6-hydroxydopamine (6-OHDA), improved mitochondrial function, and increased intracellular ATP levels [43], while also restoring mitochondrial function in SN4741 cells exposed to the mitochondrial toxin N-methyl-4-phenylpyridinium (MPP^+^) [44]. Furthermore, myricitrin showed neuroprotective effects against 6-OHDA-induced neurodegeneration in vivo [45] and reduced oxidative stress and p53 upregulation in traumatic spinal cord injury [46].

To address the lack of prior research on the effects of myricitrin in the presence of copper ions, we aimed to investigate its impact on the survival of SH-SY5Y neuroblastoma cells exposed to toxic copper concentrations and to explore the cellular and molecular mechanisms involved. Our findings reveal that myricitrin exacerbates copper-induced toxicity, largely through the activation of the ROS/p53/p38 signalling cascade and an associated increase in intracellular Ca^2+^ levels.

## 2. Materials and Methods

### 2.1. Chemicals

BAPTA-AM, crystal violet, 4-diamino-2,3-dicyano-1,4-bis[2-aminophenylthio]-butadiene (U0126), 2′,7′-dichlorofluorescin diacetate (DCF-DA), ethylenediaminetetraacetic acid (EDTA), Hoechst 33342, leupeptin, nifedipine, 3-(4,5-dimethylthiazol-2yl)2,5-dyphenyl-2H-tetrazolium bromide (MTT), propidium iodide, and staurosporine were obtained from Sigma-Aldrich Chemicals (St. Louis, MO, USA). The materials for the culturing of SH-SY5Y neuroblastoma cells, including Dulbecco’s Modified Eagle’s Medium (DMEM), fetal bovine serum (FBS), trypsin, penicillin–streptomycin solution, Na-pyruvate, and L-glutamine were purchased from Sigma-Aldrich (St. Louis, MO, USA) and Gibco (Paisley, UK). Myricitrin was purchased from TCI Chemicals Pvt. Ltd. (Chennai, India). Wortmannin was obtained from Ascent Scientific (Princeton, NJ, USA). MK-801 hydrogen maleate, pifithrin-α, SP600125, SB203580, and PARP inhibitor VIII (PJ34) were purchased from Alfa Aesar (Ward Hill, MA, USA). Copper sulphate pentahydrate was purchased from Kemika (Zagreb, Croatia), whereas DMSO was obtained from Grammol (Zagreb, Croatia). All other chemicals used were of analytical grade.

### 2.2. Culturing of SH-SY5Y Cells

The cell line SH-SY5Y has been derived from the metastatic neuroblastoma tissue originating from a 4-year-old girl. It is a well-established and routinely employed in vitro model for studying the various pathophysiological aspects of neurodegenerative diseases [47]. Cells were kindly provided by Prof. G. Šimić (University of Zagreb Medical School, Croatian Institute for Brain Research, Zagreb, Croatia) and were used between passages 15–30. They were grown in high-glucose DMEM supplemented with 10% heat-inactivated FBS, 2 mM L-glutamine, and antibiotics (100 units/mL penicillin G and 100 µg/mL streptomycin) in a humidified atmosphere of 5% CO_2_ at 37 °C. Depending on the size of the well/flask, cells were seeded at a density of 62 × 10^3^ cells/cm^2^ and allowed to attach overnight. The following day, the medium was changed, and cells were treated with copper, myricitrin, and/or selected inhibitors prepared in a fresh medium. The treatment lasted for 24 h.

### 2.3. Drug Treatment

The concentrations of copper and various inhibitors were selected based on previous studies and preliminary experiments [48,49]. Briefly, a 0.5 mM concentration of CuSO_4_ was selected as it induces mild to moderate toxic injury to SH-SY5Y cells. Inhibitors of various signalling pathways were applied in those concentrations that did not affect cell viability per se. A stock solution of myricitrin (10 mg/mL) was prepared in DMSO. For dose–response studies, SH-SY5Y cells were exposed to increasing concentrations of myricitrin for 24 h. In experiments aimed to investigate the effects of myricitrin in the presence of copper, SH-SY5Y cells were treated with 0.5 mM of CuSO_4_ and non-toxic concentrations of myricitrin (1–20 µg/mL).

Solutions of copper and myricitrin were prepared as double or quadruple concentrations in medium without/with inhibitors, respectively. The appropriate dilutions of copper (0.5 mM) and myricitrin (1–20 µg/mL) were achieved after mixing the appropriate volumes of copper and myricitrin directly in the well/flask. By this procedure, we prevented eventual reaction/complexation between copper and myricitrin in the tube, prior to the contact with the cells.

To study the effects of myricitrin on the intracellular calcium channels and signalling cascades, SH-SY5Y cells were treated with the following inhibitors: wortmannin (30 nM)—inhibitor of phospatidylinositol-3-kinase (PI3K)/Akt pathway, U0126 (1 µM)—inhibitor of MAP kinase kinases, MEK1 and MEK2, used as an inhibitor of the Raf/MEK/ERK pathway, SB203580 (10 µM)—inhibitor of p38 kinase, SP6000125 (5 µM)—inhibitor of c-Jun N-terminal (JNK) kinase, pifithrin-α (0.5 µM)—inhibitor of the transcriptional activity of p53, PARP inhibitor VIII (PJ34, 5 µM)—inhibitor of the poly(ADP-ribose) polymerase (PARP), nifedipine (10 µM)—L-type calcium channel blocker, BAPTA-AM (10 µM)—a selective chelator of intracellular Ca^2+^ ions, MK-801—a selective non-competitive antagonist of N-methyl-D-aspartate (NMDA) glutamate receptor, and leupeptin (10 µM)—an inhibitor of calpains, the calcium-dependent proteases. All inhibitors were added 1 h prior to treatment with copper and myricitrin, and cells were incubated for the next 24 h.

### 2.4. Assessment of Neuronal Viability

#### 2.4.1. MTT Assay

The colorimetric MTT assay is based on the conversion of the MTT, a yellow tetrazolium salt, to purple insoluble formazan crystals by the activity of mitochondrial enzymes succinate dehydrogenase in metabolically active, live cells. The amount of formazan generated is thus directly proportional to the number of viable cells and can be quantified spectrophotometrically at 570 nm. Following 24 h of treatment, MTT solution was prepared in DMEM (final concentration 0.5 mg/mL), 40 μL of solution was added directly to cells in empty wells, and plates were incubated at 37 °C for 3 h, protected from light. Then, 160 μL of dimethyl sulfoxide (DMSO) was added to the formazan crystals produced and shaken for 5 min on an orbital shaker. The absorbance of each well was recorded by an automatic microplate reader (Multiskan MS, Labsystems, Vantaa, Finland). The background absorbance (the absorbance of cell-free medium with added MTT solution) was subtracted from all sample readings. Ultimately, the percentage of viable cells was calculated by using the following formula:

% Viability = (Mean A_sample_/Mean A_control_) × 100

A—absorbance.

#### 2.4.2. Crystal Violet Assay

The viability of SH-SY5Y cells was also assessed using a crystal violet assay, which relies on the dye’s ability to bind to cellular proteins and DNA. During cell death, cells detach from the surface of the cell culture plates. The washing procedure removes these non-adherent cells, resulting in reduced crystal violet staining in wells containing dead or dying cells [50].

For the crystal violet assay, SH-SY5Y cells were treated with copper and myricitrin in 24-well plates. After the treatment period, the medium was removed, and cells were rinsed twice with PBS. The cells were then fixed by adding 200 μL of 4% formaldehyde to each well. Following a 20 min incubation at room temperature, the formaldehyde was removed, and the wells were rinsed with PBS. Subsequently, 200 μL of 0.5% crystal violet solution was added to each well and cells were stained for 30 min at room temperature. The wells were then washed 4 to 5 times with 500 μL of dH_2_O until fully decolorized and left to dry.

Next, 330 μL of methanol was added to each well, and the plate was placed on an orbital shaker for 20 min to completely dissolve the dye. Three 100 μL aliquots were transferred to a 96-well plate and the absorbance at 570 nm was measured using the automated microplate reader (Multiskan MS, Labsystems, Vantaa, Finland). Similarly to the MTT assay, the percentage of viable cells was calculated using the following equation:

% Viability = (Mean A_sample_/Mean A_control_) × 100

#### 2.4.3. Measurement of Intracellular ATP Level

ATP also correlates with cell viability. Therefore, we assessed ATP content using the Mitochondrial ToxGlo™ Assay (Promega, Madison, WI, USA), which relies on ATP-dependent bioluminescent luciferase reaction. Briefly, after treating the SH-SY5Y cells with copper and myricitrin, 100 µL of ATP Detection Reagent containing luciferin, ATPase inhibitors, and luciferase was added to each sample well. The plate was then shaken on an orbital shaker for 2 min to induce cell lysis. Following a 10 min incubation at RT, the luminescence produced was measured using a Tecan Infinite M200 luminometer (Salzburg, Austria) with an integration time of 1000 ms. The results are expressed as percentage of ATP levels present in vehicle-treated control cells.

### 2.5. Measurement of Lactate Dehydrogenase (LDH) Release from SH-SY5Y Cells Treated with Copper and Myricitrin

The cytotoxic effect of the copper and myricitrin treatment on the plasma membrane integrity of SH-SY5Y cells was assessed by using the fluorometric CytoTox-ONE™ Homogeneous Membrane Integrity Assay (Promega, Madison, WI, USA). LDH is a stable cytoplasmic enzyme present in eukaryotic cells that catalyzes the conversion of lactate to pyruvate together with producing NADH. The assay is based on the determination of LDH activity released into the culture medium from the cells with the damaged plasma membranes because of cell death.

Following a 24 h treatment, aliquots of the culture medium (80 μL) were taken from the 96-well culture plates. The culture medium was combined with the 80 µL of assay mixture containing lactate and NAD+ in excess, driving the LDH reaction and NADH production. The NADH further participates in the diaphorase-catalyzed generation of the fluorescent resorufin product from the resazurin substate. The medium and assay mixture were incubated for 60 min at 37 °C. Ultimately, the amount of the resorufin produced is proportional to the amount of LDH released. The fluorescence was measured with an excitation wavelength of 560 nm and an emission wavelength of 590 nm (Tecan Infinite M200, Salzburg, Austria).

### 2.6. Measurement of Intracellular ROS

The intracellular production of ROS as an indicator of oxidative stress severity was investigated using the well-established cell-permeable reagent 2′,7′–dichlorofluorescin diacetate (DCF-DA) and then quantified spectrophotometrically. DCF-DA is a fluorogenic dye that diffuses into the cells, where it is deacetylated by cellular esterases to a non-fluorescent, reduced form of the compound, 2′,7′-dichlorofluorescin (DCFH). If oxidized by reactive radicals, DCFH forms highly fluorescent 2′,7′–dichlorofluorescein (DCF). The high fluorescence intensity indicates the presence of large amounts of ROS. Therefore, this method is considered a reliable indicator of overall ROS production and is appropriate for evaluating the prooxidant properties of various xenobiotics [51].

In brief, following exposure to copper and myricitrin, SH-SY5Y cells were treated with 50 μM DCF-DA solution, at 37 °C for 30 min, in darkness. The cells were then washed with PBS and incubated for an additional hour in PBS. DCF formation was detected by fluorescence spectroscopy (Tecan Infinite M200, Salzburg, Austria), using an excitation wavelength of 485 nm and an emission wavelength of 535 nm. The fluorescence intensity detected in control cells (100% FI) was used to calculate the percentage of change in cells treated with copper and myricitrin.

### 2.7. Determination of GSH/GSSG Ratio After Treatment with Copper and Myricitrin

Glutathione is the primary non-enzymatic antioxidant in eukaryotic cells, crucial for maintaining cellular redox homeostasis. It predominantly exists in its reduced thiol form (GSH), which constitutes up to 98% of total glutathione under physiological conditions [52]. The oxidized form, glutathione disulfide, GSSG, is formed when GSH is oxidized by excess ROS under pro-oxidative conditions. The γ-glutamyl bond in the GSH tripeptide has an important trapping role in detoxifying ROS moieties and reactive electrophiles. Consequently, the GSH/GSSG ratio is often used as an important biomarker for assessing the oxidative status of cells [53].

Changes in GSH and GSSG levels were measured using a luminescence-based GSH/GSSG-Glo Assay (Promega, Madison, WI, USA), following the manufacturer’s instructions. Briefly, the assay involves the conversion of Luciferin-NT to luciferin, in a GSH-dependent reaction catalyzed by glutathione-S-transferase. Therefore, the amount of luciferin produced, which generates the luminescent signal, is proportional to the GSH present in the sample.

Total and oxidized glutathione were determined in two separate reactions. In one reaction, the total glutathione (GSH and GSSG) from the cell lysate was converted to its reduced form. In a parallel reaction, only GSSG was measured because the GSH blocking agent, N-ethyl maleimide (NEM), was added to the lysis reagent, enabling only GSSG to contribute to the luminescent signal. For quantification, this blocking reaction was followed by the conversion of GSSG to GSH and the subsequent GSH-dependent luminescent reaction.

SH-SY5Y cells were exposed to copper and myricitrin for 24 h. After removing the medium, 50 μL of lysis reagent for either total glutathione analysis or oxidized GSSG analysis was added to the wells for cell lysis, and the plates were shaken for 5 min at 700 rpm. Following this, 50 μL of Luciferin Generation Reagent was added to each well, briefly shaken and incubated for 30 min at room temperature. Finally, 100 μL of Luciferin Detection Reagent was added to each well, briefly shaken again, and after a 15 min incubation at RT, the luminescence was recorded using a Tecan Infinite M200 microplate reader (Salzburg, Austria).

The GSH/GSSG ratio was calculated from the relative luminescent units (RLU) using the following equation:

GSH/GSSG ratio = (Total glutathione RLU − GSSG RLU)(GSSG RLU/2)

RLU—relative fluorescence units.

### 2.8. Determination of SOD Activity After Treatment with Copper and Myricitrin

Superoxide dismutase (SOD) is an enzyme that catalyzes the dismutation of the superoxide radical into molecular oxygen and hydrogen peroxide. As such, SOD serves as an important mechanism of anti-oxidative defence against ROS-mediated injury.

We measured SOD activity using the SOD Determination Kit (Sigma-Aldrich, St. Louis, MO, USA) that is based on the water-soluble tetrazolium salt WST-1 and xanthine oxidase (XO) reaction. XO catalyzes the oxidation of hypoxanthine to xanthine, producing superoxide radicals. When reduced by the superoxide anion, WST-1 forms a coloured formazan product. Thus, the WST-1 reduction is proportional to the amount of superoxide radicals present. As SOD inhibits the WST-1 reaction by reducing the availability of superoxide anions, the SOD activity is determined by measuring the decrease in formazan production in terms of percent inhibition.

After treatment, SH-SY5Y cells (2 × 10^6^) were harvested by trypsinization, lysed in ice-cold lysis buffer (0.1 M Tris/HCl with 0.5% Triton X-100 and 5 mM β-mercaptoethanol), sonicated, and centrifuged at 13,200× *g* for 8 min at +4 °C. The supernatant was collected, and reactions were prepared in a 96-well plate by adding appropriate aliquots of the sample, WST working solution, enzyme working solution, and dilution buffer according to the manufacturer’s instructions. The plate was shaken thoroughly and incubated at 37 °C for 20 min. The absorbance at 450 nm was recorded using an automated microplate reader (Multiskan MS, Labsystems, Vantaa, Finland).

SOD activity (inhibition rate %) was calculated using the following equation:

SOD Activity (inhibition rate %) = ((A_blank1_ − A_blank3_) − (A_sample_ − A_blank2_)/(A_blank1_

− A_blank3_)) × 100

A = absorbance at 450 nm.

blank1—no sample added.

blank2—no enzyme working solution added.

blank3—no sample and enzyme working solution added.

### 2.9. Determination of Caspase-3/7 Activity

Caspase-3/7 are key effector enzymes in the process of caspase-dependent apoptosis which may be activated under pro-oxidative conditions. Caspase-3/7 activity was determined by using the Caspase-Glo 3/7 Assay (Promega, Madison, WI, USA). The principle of the assay is based on the use of a luminogenic substrate that, when cleaved by active caspases, produces a light proportional to the caspase-3/7 activity.

The aminoluciferin substrate from the assay contains the tetrapeptide sequence DEVD which is specifically cleaved by caspases-3/7, releasing the aminoluciferin. The aminoluciferin then serves as a substrate for luciferase which catalyzes the production of light (luminescence). The amount of luminescence generated is proportional to the amount of aminoluciferin released.

The assay was performed by directly adding 90 µL of Caspase-Glo 3/7 reagent to cells in a 96-well plate and shaken with an orbital shaker at 500 rpm for 30 s to accomplish cell lysis. The plate was then incubated at room temperature for 90 min. The luminescent signals were recorded by Tecan Infinite M200 microplate reader (Salzburg, Austria). The blank reaction was used to measure background luminescence associated with the cell culture system and Caspase-Glo^®^ 3/7 Reagent. The value of the blank reaction was subtracted from experimental values.

### 2.10. Nuclear Staining with Hoechst 33342 and Propidium Iodide (PI)

To better characterize the type of death induced by copper and myricitrin exposure, we looked for changes in chromatin morphology. Cells were stained with 1 µg/mL of Hoechst 33342 and 1 µg/mL PI for 5 min in the dark, directly in the tissue culture dish in the DMEM. The photographs were taken with the EVOS FLoid Cell Imaging Station (Invitrogen, Waltham, MA, USA). A quantitative analysis was performed by counting more than 500 cells from photographs obtained in three separated experiments.

### 2.11. Western Blot Method

After treatment with copper and myricitrin, whole-cell lysates were prepared by scraping cells into PBS containing protease inhibitors (Complete, Mini, EDTA-free protease inhibitor cocktail tablets; Roche, Indianapolis, IN, USA) and sonicating them with a 1 mm probe for 2 × 15 s. The protein concentration was measured using the Pierce BCA Protein assay (Thermo Fisher Scientific, Rockford, IL, USA). Protein samples (30 μg) were separated on 12% SDS-PAGE, transferred to nitrocellulose membranes overnight, and blocked with 5% nonfat milk in TBST for 30 min. Membranes were incubated overnight with primary antibodies, followed by secondary antibodies. Bands were visualized with chemiluminescence (Western Lightning Plus-ECL Enhanced Chemiluminescence Substrate, PerkinElmer, Waltham, MA, USA) and quantified using ImageJ 2.1.0 software following detection with the Alliance 4.7 imaging system (UVItec Cambridge, London, UK).

The antibodies used were anti-PARP-1 (F-2: sc-8007, Santa Cruz Biotechnology, 1:1000), anti-p53α (1801: sc-98, Santa Cruz Biotechnology, 1:1000), anti-PUMA (G-3: sc-374223, Santa Cruz Biotechnology, 1:1000), anti-p73 (ab40658, Abcam, 1:3000), anti-Bcl-2 (C-2: sc-7382, Santa Cruz Biotechnology, 1:1000), anti-Bax (B-9: sc-7480, Santa Cruz Biotechnology, 1:500), anti-NME1/NME2 (homemade, provided by I. Lascu, Bordeaux, France and S. Volarević, Rijeka, Croatia, 1:3000), and β-actin (7D2C10, 60008-1-Ig, Proteintech; 1:10,000).

### 2.12. Atomic Force Microscopy (AFM) Measurements

AFM cell imaging, surface roughness, and force measurements were performed using a MultiMode Scanning Probe Microscope with a Nanoscope IIIa Controller (Bruker, Billerica, MA, USA), equipped with a 125 µm Vertical Engagement (JV) scanner. An optical camera was connected to the AFM to accurately position the AFM probe above the sample. Prior to each measurement, the liquid cell (Bruker, Billerica, MA, USA) was thoroughly cleaned with ultrapure water and ethanol. All measurements were performed using the same triangular tip (MSNL, Bruker), with a spring constant *k*_nom_ = 0.07 N m^−1^ and a nominal frequency *f*_nom_ = 22 kHz. During imaging and force measurements, the temperature was maintained at 37 °C using a Digital Instruments High Temperature Heating Controller, with a resolution of 0.1 °C and an accuracy of 3%.

To ensure data accuracy, both trace and retrace images were recorded and compared during scanning. Additionally, to prevent any potential influence of the applied force on the imaging of the different central cell regions, detailed images of 2 μm × 2 µm were acquired under optimized imaging conditions. The spring constant of the cantilever was calibrated using a reference cantilever after piezo-sensitivity curves were recorded for each sample. Approximately 480 points were measured at the central part of the cells at random locations across the cell surface to average slight variations and obtain representative data. Elasticity was extracted from the force measurements following the method described by Roa et al. [54].

The glass plate containing the cell samples in a Petri dish was sealed to the standard sample holder by rubber adhesive. For each neuroblastoma cell group, we analyzed 7–12 cells (*N*cell = 7–12) from two independent experiments. Images were processed using NanoScopeTM software (Digital Instruments, version V614r1). All images are presented as raw data, except for the two-dimensional first-order attenuation.

## 3. Results

### 3.1. Treatment with Myricitrin Increases Toxic Effect of Copper on SH-SY5Y

**Figure 1:**
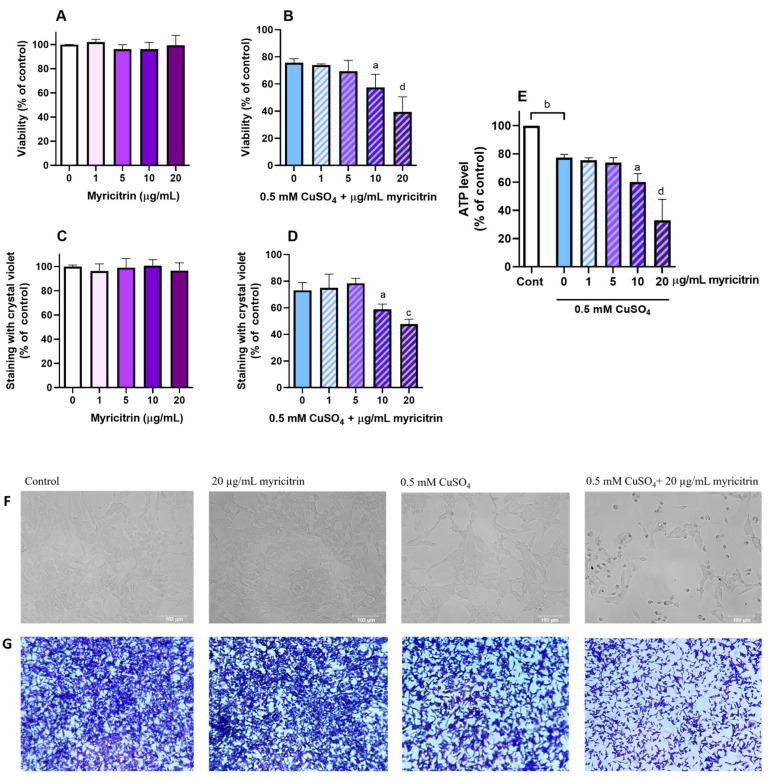
Effect of myricitrin on the viability of SH-SY5Y cells under physiological conditions and following exposure to excess copper ions. SH-SY5Y cells were treated with myricitrin at concentrations of up to 20 µg/mL for 24 h. Cell viability was assessed using the MTT assay (**A**,**B**), crystal violet staining (**C**,**D**), and ATP content measurement (**E**). Morphological changes in SH-SY5Y cells were observed using the EVOS Floid Cell Imaging System (**F**) and were also photographed following crystal violet staining (**G**). Statistical analysis was performed using one-way ANOVA followed by Dunnett’s multiple comparison tests. ^a^ *p* < 0.05, ^b^ *p* < 0.01, ^c^ *p* < 0.001, and ^d^ *p* < 0.0001 vs. 0 group (0.5 mM CuSO_4_). Data are presented as means ± standard deviation from 3 to 4 independent experiments (one-way ANOVA followed by post hoc Dunnett’s test).

We studied the effects of myricitrin on the survival of neuroblastoma SH-SY5Y cells exposed to excess copper ions. When applied alone, myricitrin did not affect cell viability at concentrations up to 20 µg/mL, as assessed by the MTT assay (Figure 1A) and crystal violet staining (Figure 1B). Exposure to 0.5 mM CuSO_4_ alone decreased cell survival to 74.79 ± 3.3% (mean ± SD) compared to the control group (Figure 1C). The simultaneous treatment of SH-SY5Y cells with copper and the non-toxic concentrations of myricitrin significantly enhanced copper’s toxic effects. The MTT assay results (Figure 1C) showed that myricitrin at concentrations of 10 and 20 µg/mL further reduced cell survival to 57.4% and 39.4%, respectively (Dunnett’s test following one-way ANOVA).

The toxic effect of myricitrin in the presence of copper was further validated by crystal violet staining and the measurement of ATP content. Crystal violet staining showed that myricitrin at concentrations of 10 and 20 µg/mL had no toxic effects when applied alone but enhanced the toxicity induced by copper ions. In comparison to cells treated with 0.5 mM CuSO_4_ alone, where 74.8% of cells remained adherent, the percentage of adherent cells decreased to 58.8% and 47.8% in the presence of 10 and 20 µg/mL myricitrin, respectively (Figure 1D). Measuring intracellular ATP levels is yet another method for assessing metabolic activity and cell viability [55]. Treatment with 0.5 mM CuSO_4_ reduced intracellular ATP levels to 77.4% of the control (*p* < 0.01, Dunnett’s multiple comparisons test following one-way ANOVA). In the presence of 10 and 20 µg/mL myricitrin, intracellular ATP levels were further reduced to 60.0% and 32.9% of the control, respectively (*p* < 0.05 for 10 µg/mL myricitrin and *p* < 0.0001 for 20 µg/mL myricitrin; one-way ANOVA, Dunnett’s test) (Figure 1E). The toxic effect of myricitrin was also evident under a light microscope (Figure 1F,G).

### 3.2. Myricitrin Enhances ROS Production While Reducing GSH/GSSG Ratio and SOD Activity

**Figure 2:**
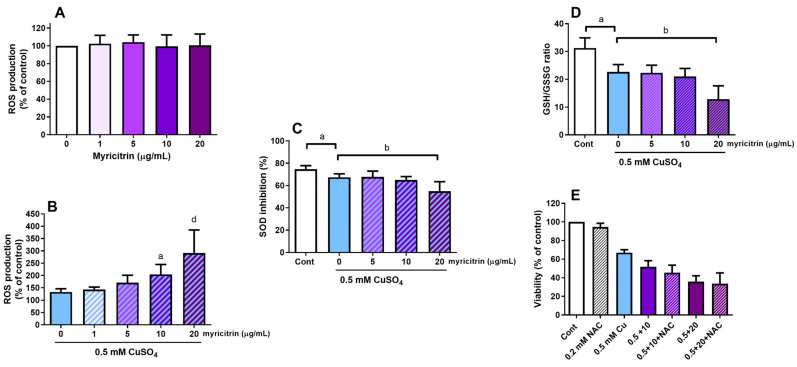
Effect of myricitrin on ROS production, SOD activity, and GSH/GSSG ratio. SH-SY5Y cells were treated with 1–20 µg/mL myricitrin alone or in the presence of 0.5 mM CuSO_4_. After 24 h, ROS levels were measured using 2′,7′-dichlorodihydrofluorescein diacetate (**A**,**B**). SOD activity was determined using a colorimetric assay. The rate of the reduction in superoxide anions in a reaction with molecular oxygen is related to xanthine oxidase activity that is inhibited by SOD (**C**). The GSH/GSSG ratio was determined using a luminescence-based system involving glutathione-S-transferase coupled to a luciferase reaction (**D**). Effect of N-acetylcysteine, a GSH precursor, on cell survival in SH-SY5Y treated with copper and 10 or 20 µg/mL myricitrin was determined by MTT assay (**E**). Results are presented as mean values ± standard deviation from 3 to 5 independent experiments performed in triplicate. ^a^ *p* < 0.05, ^b^ *p* < 0.01, and ^d^ *p* < 0.0001 compared to copper-only treatment (one-way ANOVA followed by post hoc Dunnett’s test for (**A**,**B**,**D**,**E**); Student’s *t*-test for (**C**)).

Since the toxic effects of copper are mediated by increased ROS production and oxidative stress induction, we investigated whether myricitrin influences ROS accumulation in SH-SY5Y cells. As shown in Figure 2A, myricitrin alone did not significantly affect ROS levels. However, in the presence of copper, myricitrin showed prooxidant activity, increasing the intracellular accumulation of ROS. Compared to the copper-only group, ROS production increased by 53.7% (*p* < 0.05) and 118.9% (*p* < 0.0001) compared to the copper group, at myricitrin concentrations of 10 and 20 µg/mL, respectively (Figure 2B). We also determined the activity of SOD, a key antioxidant enzyme. Treatment with copper alone slightly but significantly inhibited SOD activity by 9.7%. In the presence of 20 µg/mL myricitrin, SOD activity was further reduced by 18.5% (Figure 2C). Next, we evaluated the ratio of reduced (GSH) to oxidized (GSSG) glutathione. Treatment with 0.5 mM CuSO_4_ decreased the GSH/GSSG ratio from 31.3 to 22.7, a reduction of 27.5%. This decrease was further amplified by the addition of 20 µg/mL myricitrin, resulting in a GSH/GSSG ratio of 12.9 (Figure 2D). To investigate whether N-acetylcysteine (NAC), a precursor for GSH synthesis, could improve the survival of SH-SY5Y cells exposed to copper and myricitrin, we tested the two highest concentrations of myricitrin, which had the most pronounced effect on copper toxicity. However, NAC supplementation (0.2 mM) did not improve the viability of SH-SY5Y neuroblastoma cells under these conditions (Figure 2E).

### 3.3. The Effect of Copper and Myricitrin on Caspase Activation and Chromatin Condensation

**Figure 3:**
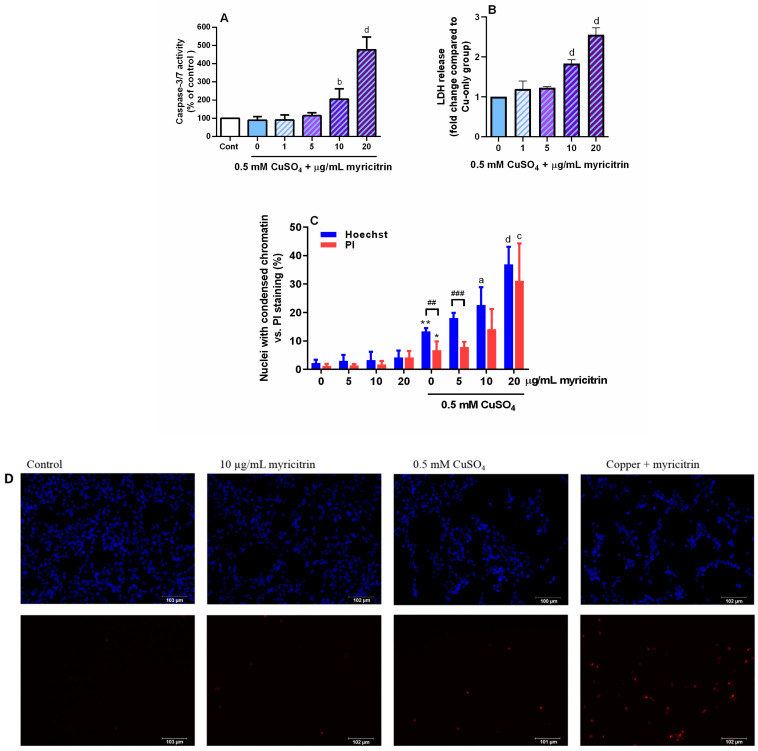
Effects of copper and myricitrin co-treatment on caspase-3/7 activity, membrane integrity, and nuclear changes. SH-SY5Y cells were treated with 0.5 mM CuSO_4_ and myricitrin (1–20 µg/mL), and caspase-3/7 activity was measured using a luminogenic substrate (**A**). Membrane damage was assessed by measuring LDH activity in the culture medium (**B**). Chromatin condensation was assessed using Hoechst 33342 staining, while propidium iodide staining was used to detect cells in late apoptosis/necrosis (**C**). Data are presented as mean values ± standard deviation from three independent experiments performed in duplicate (**A**,**C**) or triplicate (**B**). * *p* < 0.05, ** *p* < 0.01 vs. control group; ^a^ *p* < 0.05, ^b^ *p* < 0.01, ^c^ *p* < 0.001, and ^d^ *p* < 0.0001 vs. copper alone (one-way ANOVA followed by post hoc Dunnett’s test) and ^##^ *p* < 0.01, ^###^ *p* < 0.01 (Student’s *t*-test). Representative images obtained by staining with Hoechst 33342 (blue) and propidium iodide (red) were taken using the EVOS Floid Cell Imaging System (**D**).

Since oxidative stress can trigger different forms of cell death depending on the severity of damage and the activation of specific intracellular events, we aimed to better characterize the characteristics of SH-SY5Y cell death following copper and myricitrin treatment. Apoptosis, a form of programmed cell death, can occur via caspase-dependent or caspase-independent pathways, both of which are associated with nuclear changes such as chromatin condensation and DNA fragmentation [56]. As caspases 3 and 7 are the key mediators of classical apoptosis, we assessed their activation following exposure to copper and myricitrin. After 24 h of treatment with 0.5 mM CuSO_4_, caspase activity remained comparable to that observed in the control group. However, co-treatment with myricitrin significantly increased caspase-3/7 activity by 105.6% and 376.9% at 10 µg/mL and 20 µg/mL myricitrin, respectively (Figure 3A). To further assess cell membrane integrity, we measured LDH activity in the culture medium. Compared to the copper-only group, co-treatment with myricitrin resulted in a greater release of cytoplasmic LDH, indicating more pronounced membrane damage. Compared to copper alone, myricitrin at 10 and 20 µg/mL further increased LDH activity by 83.5% and 155.4%, respectively (Figure 3B). Next, we investigated changes in chromatin condensation using the fluorescent dye Hoechst 33342. The percentage of cells with bright, condensed nuclei increased from 2.3% to 13.4% in SH-SY5Y cells exposed to 0.5 mM CuSO_4_. Co-treatment with 10 and 20 µg/mL myricitrin further increased these percentages to 22.6%, and 37.0%, respectively. Additionally, we used propidium iodide (PI) staining to detect cells in late-stage apoptosis or undergoing necrosis. Copper treatment increased the percentage of PI-positive nuclei from 1.2% to 6.8%, and this was further enhanced in cells co-treated with 20 µg/mL myricitrin (Figure 3C).

### 3.4. Effects of Myricitrin on the Expression of Proteins Involved in Oxidative Stress Response and Cell Death in SH-SY5Y Cells Exposed to Excess Copper

**Figure 4:**
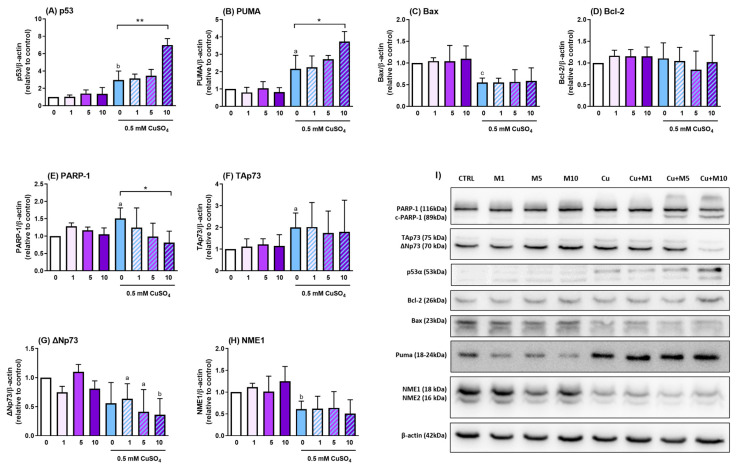
Effects of copper and myricitrin on the expression of apoptotic and oxidative stress-related proteins. Protein expression levels of p53 (**A**), PUMA (**B**), Bax (**C**), Bcl-2 (**D**), PARP-1 (**E**), TAp73 (**F**), ΔNp73 (**G**), and NME1 (**H**) were assessed 24 h after exposure to 0.5 mM CuSO_4_ and/or 1, 5, and 10 µg/mL myricitrin. Total cellular proteins were extracted, separated by polyacrylamide gel electrophoresis, and transferred to nitrocellulose membranes. The membranes were incubated with specific primary antibodies, and subsequently with horseradish peroxidase-conjugated secondary antibodies. Protein bands were visualized using enhanced chemiluminescence. β-actin was used as a loading control to normalize protein expression levels. Data are presented as mean ± SD from 3 to 4 independent experiments. Densitometric analysis was performed using ImageJ (Fiji) software. Data were analyzed using an unpaired *t*-test (* *p* < 0.05, ** *p* < 0.01 vs. copper group; ^a^ *p* < 0.05, ^b^ *p* < 0.01, and ^c^ *p* < 0.001 vs. control group). Representative Western blot images are shown in (**I**).

To better understand the molecular mechanisms underlying the toxic effects of myricitrin in the presence of excess copper, we examined changes in the expression of key proteins involved in cell death regulation and oxidative stress response. The transcription factor p53 plays a crucial role in orchestrating the cellular response to oxidative stress and DNA damage. Copper exposure alone significantly increased p53 levels, while co-treatment with 10 µg/mL myricitrin further elevated p53 expression, reaching nearly 7 times higher values compared to the control group (Figure 4A). PUMA and Bax, pro-apoptotic proteins usually regulated by p53, responded differently to copper and myricitrin. Copper alone increased PUMA expression by 2.2-fold, and the addition of 10 µg/mL myricitrin further enhanced PUMA levels by 72% compared to the copper group (Figure 4B). On the contrary, Bax, which is directly involved in the mitochondrial pathway of apoptosis, was reduced following exposure to copper alone, and remained unchanged in the presence of all tested concentrations of myricitrin (Figure 4C). Although p53 can affect neuronal death by modulating levels of the pro-survival protein Bcl-2, we did not observe any significant changes in Bcl-2 expression across all treatment groups (Figure 4D). Poly(ADP-ribose) polymerase 1 (PARP-1) is a key enzyme involved in DNA damage repair, particularly during oxidative stress, and can be cleaved by activated caspases-3 and -7. Following copper treatment, an increase in PARP-1 expression was observed. Treatment with myricitrin reversed this copper-induced upregulation of the full-length PARP-1 and also promoted its cleavage, resulting in the generation of an 89 kDa fragment (Figure 4E). We also analyzed the expression of p73, a member of the p53 family that regulates neuronal death through its various isoforms. In copper-treated SH-SY5Y cells, TAp73 expression was upregulated, but no further increase was observed following myricitrin treatment (Figure 4F). For the ΔNp73 isoform, there was a slight but non-significant decrease in expression after copper treatment, followed by an additional non-significant decrease in cells exposed to myricitrin compared to the copper-only group. However, compared to control cells, ΔNp73 expression was significantly reduced in cells treated with copper and 1, 5, and 10 µg/mL myricitrin (*p* = 0.0335, 0.0246, and 0.038, respectively) (Figure 4G). Nucleoside diphosphate kinase NME1, which is also involved in the regulation of apoptosis and protection against oxidative damage, was reduced in response to copper treatment. Its levels did not change further in cells treated with myricitrin (Figure 4H). Similar was observed for NME2, a NME1 close relative.

### 3.5. Intracellular Calcium Mediates Neurotoxic Effects of Myricitrin

**Figure 5:**
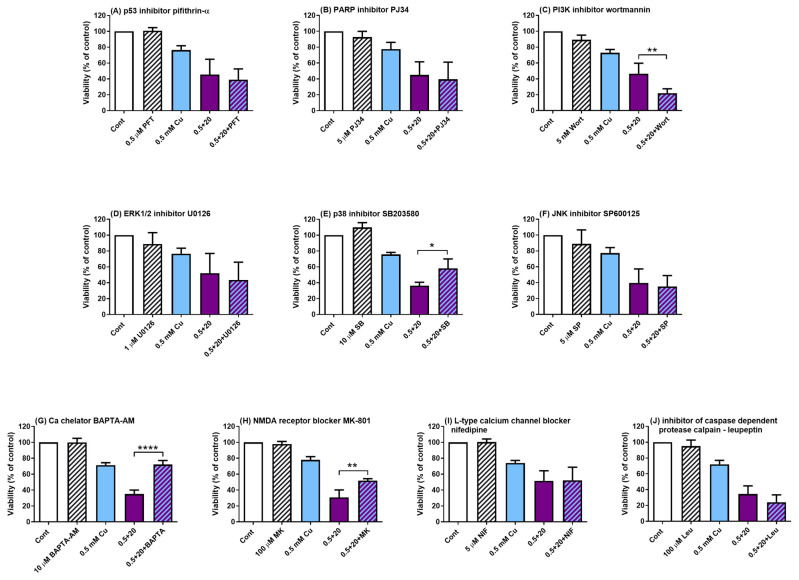
Effects of specific inhibitors and modulators of intracellular signalling pathways on the neurotoxic effect of myricitrin. Cells were treated simultaneously with 0.5 mM CuSO_4_ and 20 µg/mL myricitrin in combination with the following inhibitors: pifithrin-α (p53 inhibitor) (**A**), PJ34 (PARP inhibitor) (**B**), wortmannin (PI3K/Akt inhibitor) (**C**), U0126 (ERK1/2 inhibitor) (**D**), SB203580 (p38 inhibitor) (**E**), SP600125 (JNK inhibitor) (**F**), BAPTA-AM (intracellular calcium chelator) (**G**), MK-801 (NMDA receptor channel blocker) (**H**), nifedipine L-type calcium channel inhibitor) (**I**), and leupeptin (calpain inhibitor) (**J**). Inhibitors were applied 1 h prior to and during the 24 h treatment with copper and myricitrin. Data are presented as means ± SD from 3 to 5 independent experiments performed in triplicate. * *p* < 0.05, ** *p* < 0.01, and **** *p* < 0.0001 vs. copper + myricitrin treatment (one-way ANOVA followed by post hoc Tukey’s test).

Intracellular signalling pathways are commonly activated by oxidative stress and involved in the induction of cell death. Therefore, specific inhibitors of signalling pathways involved in oxidative stress response and cell survival were used to better elucidate the molecular mechanisms underlying the neurotoxic effects of myricitrin. Pifithrin-α, an inhibitor of transcriptional p53 activity, did not affect cell survival in SH-SY5Y cells co-treated with copper and myricitrin (Figure 5A). The same was observed for PARP inhibitor VIII (PJ34) (Figure 5B). Next, we studied the contribution of the PI3K/Akt pathway on cell viability by using the PI3K inhibitor wortmannin. Wortmannin significantly reduced the cell survival when combined with myricitrin and copper (*p* < 0.01, Tukey’s multiple comparisons test), suggesting that PI3K/Akt activation usually serves a protective role in cellular response to oxidative injury (Figure 5C). The contribution of the MAPKs signalling pathways to the toxic effect of myricitrin was examined by using the following inhibitors: U0126 (MEK1/2, i.e., ERK1/2), SB203580 (p38), and SP600125 (JNK). Except for the protective effects of the p38 pathway inhibition in cells treated with 20 µg/mL myricitrin and copper (*p* < 0.05), there were no significant effects of other inhibitors on the survival of SH-SY5Y cells (Figure 5D–F). Since calcium ions may play an important role in the activation of cell death pathways, we further evaluated the effects of calcium-modifying inhibitors. The calcium chelator BAPTA-AM significantly reduced the toxic effects of myricitrin (*p* < 0.0001, Figure 5G). The effects of MK-801 (NMDA receptor blocker) and nifedipine (L-type calcium channel blocker) were also studied. Co-treatment with MK-801 improved the viability of SH-SY5Y cells (*p* < 0.01, Figure 5H), while nifedipine had no effect at both concentrations of myricitrin (Figure 5I). Finally, the role of calpain inhibition in cell survival was assessed using leupeptin. However, the addition of 100 µM leupeptin did not influence the viability of SH-SY5Y cells treated concomitantly with copper and myricitrin (Figure 5J).

### 3.6. Effects of Myricitrin and Copper on the Morphological Appearance of SH-SY5Y Cells at the Nanoscale

**Figure 6:**
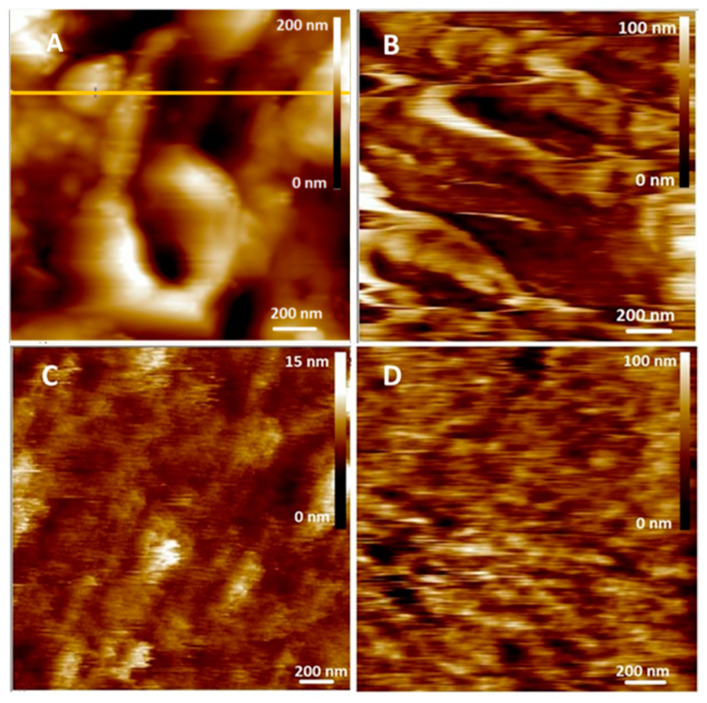
2D height images of SH-SY5Y neuroblastoma cells using AFM: (**A**) control cell; (**B**) cell treated with 10 μg/mL myricitrin for 24 h; (**C**) cell treated with copper (0.5 mM CuSO_4_) for 24 h; (**D**) cell treated with myricitrin and copper (same concentrations as in the individual treatment) for 24 h. The scan area is 2 μm × 2 μm, with vertical scales noted on the images.

**Figure 7:**
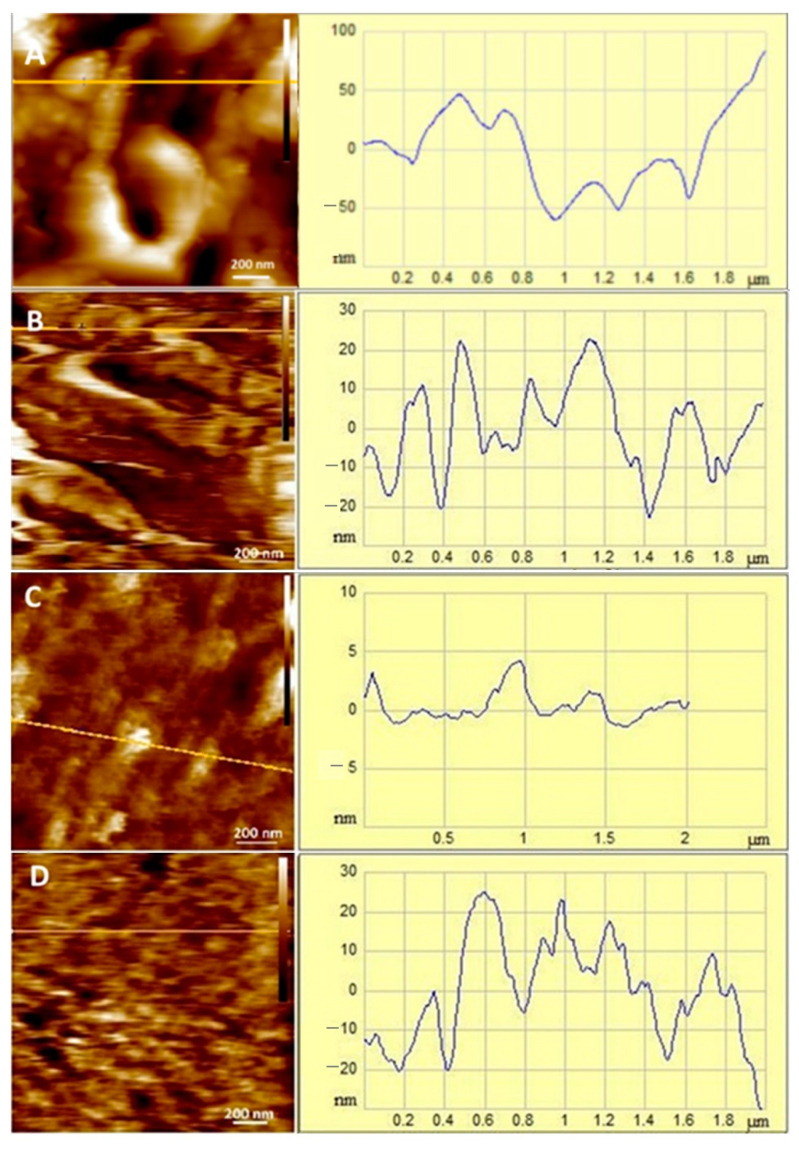
2D height images (**left**) and height section profiles along the yellow line on the height image (**right**) of neuroblastoma SH-SY5Y cells using AFM: (**A**) control cell without treatment; (**B**) cell treated with 10 μg/mL myricitrin for 24 h; (**C**) cell treated with copper (0.5 mM CuSO_4_) for 24 h; (**D**) cell treated with myricitrin and copper (same concentrations as in the individual treatment) for 24 h.

**Figure 8:**
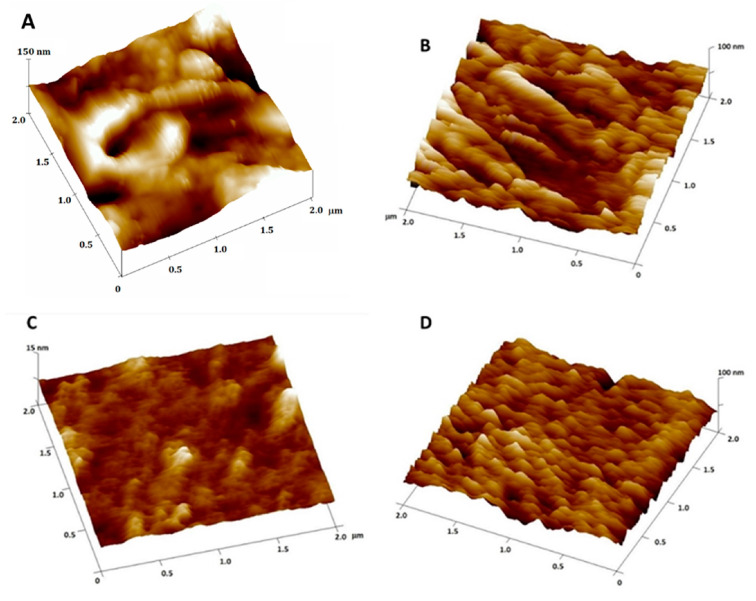
3D height images (**left**) and height section profiles along the yellow line on the height image (**right**) of neuroblastoma SH-SY5Y cells using AFM: (**A**) control cell without treatment; (**B**) cell treated with 10 μg/mL myricitrin for 24 h; (**C**) cell treated with copper (0.5 mM CuSO_4_) for 24 h; (**D**) cell treated with myricitrin and copper (same concentrations as in the individual treatment) for 24 h.

The morphological changes in SH-SY5Y neuroblastoma cells induced by treatment with 10 μg/mL myricitrin, 0.5 mM CuSO_4_, and both myricitrin and copper were examined in detail using atomic force microscopy (AFM) in both imaging and non-imaging modes. Representative AFM height images and the corresponding cross-sectional height profiles are shown in Figure 6, Figure 7 and Figure 8. In addition to the high-resolution 2D and 3D height images and cross-sectional profiles, detailed topographical information was obtained through roughness parameters: *Z*-range, *R*q, and *R*a. The *Z*-range represents the maximum distance between the highest and lowest points on the scanned surface, *R*a denotes the average roughness, while *R*q indicates the root mean square roughness value.

On the height images, relatively clear and distinct protrusions were observed on the control cells, indicating the presence of organized, soft, separated filamentous structures (Figure 6A and Figure 8A), up to 50 nm high (see section analysis in Figure 7A). The filamentous structures, which are inhomogeneously distributed across the scanned area, form depressions up to 55 nm deep in the interstices. The separated filament with homogenous distribution over the analyzed cell surface and a somewhat rougher surface were observed in our previous study [48], after the treatment of SH-SY5Y cells with myricetin. The slight difference in roughness between this and the previous experiments could be explained by the AFM measurements. Even though the AFM measurements were performed under the same conditions, the sharpness, size, and radius of curvature of each new probe used are different, which results in an exclusion of some surface features and slightly lower resolution. However, all the measurements presented in this paper were performed with the same probe and the comparison of the data obtained by comparing each treated cell sample with the control group ensures the high accuracy of the observed data [57].

However, upon treatment with myricitrin, the filamentous structures between the cells appeared more prominently, and large depressions with a depth of up to 20 nm were formed (Figure 7B). Due to the adhesion of the myricitrin within the depressions between the filamentous structures formed, the cell surface was less rough compared to the control cells (Figure 7A,B for control and myricitrin-treated cells, respectively). In cells treated with copper, filamentous structures were arranged more homogeneously and reached heights of up to 5 nm (Figure 7C). These protrusions, evenly distributed over the entire surface, indicate their ability to self-aggregate in the presence of copper ions. When SH-SY5Y cells were treated simultaneously with myricitrin and copper (Figure 7D), a rougher surface was observed over nearly the entire area (95%) (Figure 7D). The rhamnose fragment in myricitrin’s structure likely increases its adherence to the cell surface, leading to the disruption of homogeneous filamentous structures (Figure 7D), indicating the toxic effect of myricitrin in the presence of copper.

Additional data on the morphology of SH-SY5Y neuroblastoma cells were obtained by calculating the surface roughness of treated cells from eight selected 4 µm^2^ regions based on height image data (Table 1). The roughness analysis revealed that the cell surface roughness varied significantly across different treatments (Table 1). Compared to control cells, cells treated with 10 μg/mL myricitrin displayed significantly decreased roughness but higher Z-range values (Z-range = 103 ± 24 nm for control and 116 ± 40 nm for myricitrin-treated cells). The observed increase in Z-range can be attributed to myricitrin’s high affinity for adsorbing onto the cell surface, likely due to its rhamnose fragment, resulting in surface depressions and protrusions. In contrast, cells treated with copper displayed a notably smoother surface compared to control cells. However, in cells treated with both myricitrin and copper, the roughness parameters confirmed the myricitrin’s toxic effect in the presence of copper, as reflected by the consistent increase in roughness parameters compared to control cells, particularly in Z-range. While the *R*a and *R*q values for cells treated with both myricitrin and copper increased by 42% and 45%, respectively, compared to myricitrin-only treatment (Table 1), the Z-range increased by 60%, indicating the formation of aggregate-like structures. Furthermore, in copper-only treated cells, the absence of such aggregate-like structures was associated with lower *Z*-range, *R*q, and *R*a values compared to control cells, showing a decrease of 64%, 76%, and 79%, respectively.

### 3.7. Effects of Myricitrin and Copper on the Nanomechanical Properties of Neuroblastoma SH-SY5Y Cells

**Figure 9:**
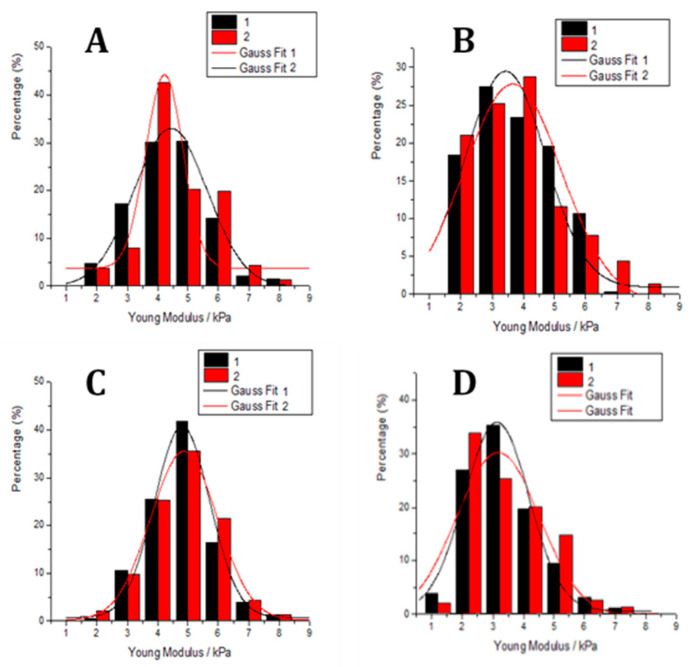
Elasticity histograms of neuroblastoma SH-SY5Y cells from two independent experiments: (**A**) control cells, (*N*_cell_ = 10), *N*_fc_ = 580; (**B**) cell treated with 10 μg/mL myricitrin for 24 h, (*N*_cell_ = 9), *N*_fc_ = 580; (**C**) cells treated with copper (0.5 mM CuSO_4_) for 24 h, (*N*_cell_ = 12), *N*_fc_ = 580; (**D**) cells treated with myricitrin and copper for 24 h, (*N*_cell_ = 7), *N*_fc_ = 580. Histograms were fitted with the Gauss function, labelled by red and black lines for each measurement.

In addition to AFM imaging and roughness analysis that can provide valuable indicators of significant cytoskeletal reorganization and filament structure within cells, measurements of the cells’ nanomechanical properties, particularly elasticity, offers further insights into the relationship between roughness parameters and the nanomechanics of neuroblastoma cells. Therefore, we performed experiments on their nanomechanical properties, i.e., elasticity, to gain a deeper insight into the correlation between the roughness parameters and the nanomechanics of the neuroblastoma cells. The key elasticity parameter determined in the nanomechanical experiments is the elastic modulus (elasticity as a measure of cell hardness). Young’s moduli were calculated from 580 force–distance curves (*N*fc) measured on control neuroblastoma cells (*N*cell = 8), with results displayed in elasticity histograms (Figure 9). The same procedure was repeated in another independent experiment, and Young’s moduli were derived by Gaussian function fitting (see Figure 9). Across all samples, elasticity values were relatively consistent, with changes in +14.25%, −19.23%, and −21.9% for cells treated with myricitrin, copper, and both myricitrin and copper, respectively, compared to control cells. Figure 9 and Table 1 summarize the elasticity histogram data from the force–distance curves (*N*fc = 580 for each treatment group).

Following treatment with 10 µg/mL myricitrin, the average Young’s modulus increased from *E* = (4.21 ± 0.17) kPa in controls to *E* = (4.81 ± 0.06) kPa in myricitrin-treated cells (Figure 9B), indicating that myricitrin-treated cells were stiffer than control cells, likely due to myricitrin adsorption on the neuroblastoma cell surface. In contrast, copper treatment reduced the Young’s modulus to *E* = (3.4 ± 0.2) kPa, representing a nearly 20% decrease, indicating that copper-treated cells were softer and had undergone significant cytoskeletal reorganization, underscoring copper’s toxic effect on cytoskeleton structure and membrane surface roughness. When cells were treated with both copper and myricitrin, the elastic modulus further decreased slightly to *E* = (3.29 ± 0.08) kPa. The observed decrease in elasticity in treated cells indicates notable cytoskeletal reorganization and further supports the neurotoxic impact of this treatment.

## 4. Discussion

We studied the effects of myricitrin, a flavonoid belonging to a flavonol group that has shown antioxidant and protective effects in previous studies [43,44,45,46,58,59]. We found that myricitrin exacerbated the detrimental effects of copper on cell survival, and lead to a further decrease in viability when applied at concentrations that were not toxic per se. This decrease was demonstrated by using MTT assay, crystal violet staining, and the measurement of intracellular ATP levels. Similarly to myricitrin, flavonols myricetin [48] and quercetin [35], as well as polyphenol-rich propolis [49], have shown neurotoxic effects in the copper-enriched environment, marked by increased ROS production and oxidative stress. Resveratrol also exhibited pro-oxidative effects in copper’s presence [60].

The main ROS produced by copper are hydroxyl radicals and superoxide anions. Hydroxyl radicals are formed in a Fenton-like reaction between Cu^+^ ions and hydrogen peroxide, generating Cu^2+^ and hydroxyl radicals. Hydroxyl radicals are the most potent and dangerous oxidants that cause significant biological damage by randomly attacking nearby DNA, lipids, and proteins [61]. In addition, Cu^+^ can catalyze a reaction with molecular oxygen, producing superoxide anions. While superoxide anions are less reactive and typically neutralized by SOD, they can still cause oxidative damage to vital cellular components. In the presence of superoxide anions, Cu^2+^ can be reduced back to Cu^+^, allowing repeated cycles of hydrogen peroxide decomposition and hydroxyl radical production [6]. More importantly, Cu^2+^ can trigger the oxidation of the hydroxyl groups of flavonoids. Redox cycling between copper ions and flavonoids also involves the generation of hydroxyl radicals and superoxide anions. The redox process begins with copper in its oxidized form (Cu^2+^) interacting with flavonoids. The flavonoid donates an electron to Cu^2+^, reducing it to its cuprous form (Cu^+^), while the flavonoid itself forms a semiquinone radical. In a reaction with molecular oxygen, unstable semiquinone radicals further yield superoxide anions and the quinone form of flavonoids, while Cu^+^ reduces superoxide anion to hydrogen peroxide, which in turn can form hydroxyl radicals through the Fenton reaction [35,62,63]. This Cu^2+^/Cu^+^ redox cycling probably underlies the pro-oxidative action of myricitrin, leading to ROS accumulation. As mentioned previously, the redox behaviour of flavonoids is highly dependent on the number of hydroxyl groups [38]. Myricitrin has five such groups, making it susceptible to oxidation and subsequent pro-oxidative effects.

In addition to ROS increase, the pro-oxidative effect of myricitrin in SH-SY5Y cells was further supported with the reduced GSH/GSSG ratio and decreased SOD activity compared to copper alone. GSH is the primary cellular antioxidant, capable of scavenging and eliminating hydroxyl radicals and superoxide anions [64]. The further decline in the GSH/GSSG ratio in cells co-treated with copper and myricitrin supports the pro-oxidative action of myricitrin in the presence of copper ions. However, as the exogenous addition of NAC, a GSH precursor, did not improve cell viability, it is likely that GSH depletion is not a decisive event in the cell death induction. In addition, if present in excess, copper can form Cu(I)-[GSH]2 complexes which exert pro-oxidative effects by generating superoxide radicals [6,64]. This complex formation, along with redox cycling between copper and flavonoids, may contribute to the observed reduction in SOD activity in SH-SY5Y cells. SOD plays a critical role in preventing oxidative damage by neutralizing superoxide anions, and its activity often decreases under oxidative stress [65]. Moreover, the formation of these complexes further depletes GSH, potentially exacerbating copper toxicity [66].

When excessive ROS production overwhelms the cell’s antioxidant defence systems, the resulting damage often becomes too severe for repair, typically leading to cell death. To obtain better insight into the cell death mechanisms, we used DNA-binding dyes PI and Hoechst 33342. An increase in the number of cells with chromatin condensation, most likely apoptotic cells, occurred after treatment with 0.5 mM CuSO_4_, and was further increased in combination with myricitrin. A significant increase in PI staining was also observed, indicating the presence of necrotic cells or cells in the late stages of apoptosis. The number of nuclei with DNA condensation was more pronounced and observed at a lower concentration compared to PI staining, suggesting a greater representation of apoptotic events. Given that chromatin condensation is a hallmark of both caspase-dependent and caspase-independent apoptosis, we measured the activities of caspases 3 and 7 which are characteristic mediators of the caspase-dependent mitochondrial pathway. We did not observe an increase in caspase-3/7 activity in cells treated with copper alone, but cell death promoted by myricitrin was accompanied with the prominent caspase-3/7 induction. Under higher oxidative stress, caspase-dependent apoptosis prevails, and some cells likely die by necrosis due to the observed impairment of membrane integrity (LDH release). The enhanced activation of caspase-3/7 was also demonstrated in P19 neurons exposed to copper and the ethanolic extract of propolis [49]. On the other hand, caspase-independent apoptosis was initiated following treatment with copper and myricetin, an aglycon form of myricitrin [48], suggesting that molecular mechanisms triggering the cell death are rather complex and determined by specific cellular context and the flavonoid of interest.

It has been shown that copper can induce DNA base modifications and strand breaks by generating hydroxyl radicals via Fenton chemistry [67]. While myricitrin alone may intercalate into DNA [68], flavonoids generally protect against oxidative damage [69]. However, Cu(II)-myricitrin complexes show stronger DNA binding and can induce multiple DNA breaks due to Cu(II) redox cycling. It is likely that these complexes create ROS formation centres near DNA breakage sites, ultimately making Cu(II)-myricitrin complexes twice as potent in inducing cell death compared to myricitrin alone [68].

A prominent increase in p53 expression has been observed following treatment with various oxidizing agents, including copper ions [70,71]. Generally, this increase promotes neuronal death via p53 targets such as PUMA, a potent pro-apoptotic BH3-only protein [72,73]. However, the expression of PUMA can be regulated by other transcription factors as well [73,74]. Copper treatment upregulated p53 and PUMA in SH-SY5Y cells, and myricitrin further amplified these effects, strongly supporting the p53’s involvement in the cytotoxic effects of myricitrin. However, pifithrin α, an inhibitor of transcriptional p53 activity, failed to improve cell viability. This likely indicates that the effects of p53 are transcription independent in our conditions. Regarding transcription-independent mechanisms, p53 may act directly on mitochondria, interacting with anti-apoptotic proteins like Bcl-2 and neutralizing their inhibitory effect on pro-apoptotic proteins such as Bax [75]. Therefore, this results in mitochondrial outer membrane permeabilization, cytochrome c release, and the subsequent activation of the caspase cascade [34,76]. Bax is a pro-apoptotic member of the Bcl-2 family, and also a p53 target that is commonly involved in mitochondrial membrane permeabilization by forming pores in the outer mitochondrial membrane [77]. However, following copper treatment, we observed reduced levels of Bax, and no further changes in the presence of myricitrin. The levels of Bcl-2, the typical anti-apoptotic protein that restrains pro-apoptotic partners such as Bax and Bak and preserve the integrity of the mitochondrial outer membrane [78], were also unchanged in our conditions. Taken together, these findings suggest that p53 likely mediates its effects via the transcription-independent mechanism and the regulation of mitochondrial-mediated apoptosis in inducing the neurotoxic effects of myricitrin.

Poly(ADP-ribose)polymerase-1 (PARP-1) is typically activated in response to DNA strand breaks, initiating DNA repair pathways to maintain genomic integrity [79]. During caspase-dependent apoptosis, PARP-1 serves as a substrate for activated caspases 3 and 7, resulting in the formation of 89 kDa fragments that loses its DNA repairing activity [80]. In SH-SY5Y cells, treatment with myricitrin reduced the levels of full-length PARP-1 at 116 kDa, while simultaneously increasing the levels of the cleaved PARP-1 fragment at 89 kDa, suggesting that myricitrin promotes the caspase-mediated cleavage of PARP-1. This inhibition of PARP-1 activity likely prevents DNA repair, thereby contributing to cell death.

In addition to apoptosis, an increase in LDH release indicates that some cells were undergoing necrotic cell death. Although necrosis is commonly perceived as an unregulated form of cell death, certain forms of necrosis are regulated and share molecular features with apoptosis yet are mechanistically distinct. A key factor in necrotic cell death is the opening of the mitochondrial permeability transition pore (mPTP). Under specific conditions of cellular stress, such as oxidative stress or calcium overload, this pore can open, resulting in the loss of mitochondrial membrane potential, the impairment of ATP production, and bioenergetic failure. It has been shown that p53 can translocate to mitochondria and interact with cyclophilin D, which plays a critical regulatory role in the pore opening. This interaction triggers mPTP opening, resulting in mitochondrial rupture and necrosis [81]. Therefore, in addition to its role in apoptosis, the observed upregulation of p53 may also contribute to necrotic cell death.

We also investigated the expression of the main p73 isoforms, TAp73 and ΔNp73. Whereas TAp73 is involved in apoptosis, ΔNp73 promotes cell survival via p53-dependent and -independent mechanisms, and the relative ratio of these isoforms could be an important determinator of the cellular response to different types of stressors [82,83,84]. However, compared to copper alone, myricitrin did not induce significant changes in the expression of these two isoforms, suggesting that neither isoform plays a critical role in the myricitrin-induced exacerbation of cell death. Similar results were observed for the redox-regulated proteins NME1 and NME2, which may be implicated in oxidative stress response [85,86]. However, no evidence was found that these proteins were involved in myricitrin-induced cell death.

To further elucidate the molecular mechanisms underlying the cytotoxic effects of myricitrin, we employed selective inhibitors targeting signalling pathways that regulate cell survival and oxidative stress responses. MAP kinases, including ERK, JNK, and p38, can be activated by ROS and mediate signalling pathways that ultimately lead to apoptosis under oxidative stress conditions [87]. While ERK may promote survival in specific contexts, JNK and p38 are typically associated with stress responses and can promote apoptosis by regulating Bcl-2 family members at both transcriptional and post-transcriptional levels, as well as influencing p53 and caspase activation [88]. Among the inhibitors tested, only the p38 inhibitor significantly attenuated the cytotoxic effect of 20 µg/mL myricitrin, suggesting a role for the p38 pathway in neuronal cell induction. The activation of p38 may result in the phosphorylation of downstream targets involved in apoptosis [89], or it may enhance p53 activity through phosphorylation, further amplifying the pro-apoptotic signals initiated by p53 [90]. Moreover, it has been reported that oxidative signalling-mediated p53 activation primarily relies on p38 MAPK [91]. Consistent with our findings, the critical role of the ROS/p38/p53 axis has been demonstrated in various models of oxidative stress-induced neuronal apoptosis [49,89,92]. We also investigated the effect of wortmannin, a PI3K inhibitor, as the PI3K/Akt signalling pathway regulates signal transduction and mediates neuronal survival [35,93,94]. The addition of wortmannin decreased cell survival in myricitrin-treated cells. Wang et al. [95] demonstrated that wortmannin reduces GSH levels and increases oxidative stress in cultured retinal pigment epithelial cells by inhibiting Nrf2-mediated antioxidant functions. This suggests that the inhibition of the PI3K/Akt pathway in SH-SY5Y cells may enhance oxidative stress, thereby reducing cell survival.

Calcium ions (Ca^2+^) play a pivotal role in neuronal apoptosis, acting as secondary messengers in apoptotic signalling pathways, including the p38 signalling cascade, and influencing mitochondrial function while stimulating ROS production [96,97,98]. The use of BAPTA-AM, which binds intracellular calcium ions, completely prevented the cytotoxic effect of myricitrin, indicating that disrupted [Ca^2+^]_i_ homeostasis is crucial for triggering cell death. As voltage-sensitive L-type Ca^2+^ channels and N-methyl-D-aspartate (NMDA) receptor ion channels are the primary pathways for calcium entry [99], we tested the effects of MK-801, an NMDA receptor open channel blocker, and nifedipine, an L-type calcium channel blocker. While MK-801 was able to abrogate the cytotoxic effects of myricitrin, nifedipine was without effect, suggesting that NMDA-gated ion channels primarily contribute to intracellular calcium levels. The chemokine protein Cxcl12 also induced neuronal death influenced by calcium overload. Interestingly, the neurotoxic effects of Cxcl12 were attenuated by both MK-801 and nimodipine (another L-type calcium channel blocker) but only MK-801 inhibited p38 MAPK activity [100]. Other studies have also indicated that neuronal death mediated by NMDA receptors involves p38 pathway activation [101,102]. Elevated calcium levels may also activate calcium-dependent enzymes, such as proteases calpains, which regulate neuronal death by affecting proteins involved in apoptosis, autophagy, and regulated necrosis [103]. The interplay between NMDA receptors, calpains, and p38 pathway has also been reported [104]. However, treatment with leupeptin, a calpain inhibitor, did not affect cell viability in our conditions, indicating that the toxic effects of myricitrin were not mediated by calpains.

The application of AFM in neuronal studies is rapidly advancing. In neuroscience, numerous experiments utilize AFM at the nanoscale to monitor the structural and mechanical changes in cell membranes under oxidative stress conditions following treatment with various agents [105,106]. In this study, we investigated the neurotoxic effects of myricitrin using AFM in both imaging and non-imaging modes at nanoscale resolution. The imaging mode allowed us to capture high-resolution morphological images and measure the surface roughness of neuroblastoma cells, while the non-imaging mode, known as force spectroscopy, provided data on the nanomechanical properties of cells under the influence of copper and myricitrin during neurodegeneration. These AFM approaches enabled the detailed assessment of cell surface morphology and cytoskeletal changes reflecting local mechanical properties [107,108].

The observed morphological changes are likely due to the reorganization of fine filamentous structures into aggregate-like formations. Since actin filaments are the major components of the cytoskeleton, their dynamics and structural alterations reflected in nanomechanical properties like elasticity and surface roughness, are largely destabilized [109]. Increased surface roughness following exposure to myricitrin with copper indicates actin network degeneration [109], while copper and myricitrin treatments significantly decreased cell elasticity. Actin disaggregation, which lowers the mean elastic modulus, highlights the essential role of actin filaments in mechanical stability and elastic resistance [108]. Such reductions in elasticity, previously observed in cells exposed to oxidative stress, are thought to arise from disrupted cytoskeleton-plasma membrane junctions, ROS overproduction, actin network degradation, and altered membrane permeability [110,111]. Our results of imaging and non-imaging AFM measurements are consistent with cellular assays indicating the myricitrin-induced loss of membrane integrity and cell death by necrosis in the presence of copper. In addition to the AFM results on the effects of myricitrin on the morphology and nanomechanics of treated neuroblastoma cells, this study also provided deeper insights into the effects of copper alone on cell surface topography and nanomechanical properties. Structural and nanomechanical changes in neuroblastoma SH-SY5Y cells, i.e., the depletion of filamentous structures and the disruption of the original dynamics of the cytoskeleton, were induced by treatment with myricitrin in the presence of copper. A deeper understanding of the nanomechanical response may provide an alternative therapeutic route to overcome the neurodegeneration induced by copper treatment.

The prooxidant effects of copper are mainly attributed to Cu^+^ ions, which catalyze the formation of toxic hydroxyl radicals, leading to lipid peroxidation and oxidative damage to various biological targets. Free copper ions can have deleterious effects through direct and/or non-specific binding to thiol and amino groups of various cellular proteins or even to bases in nucleic acids. This binding leads to conformational changes in proteins, promotes their self-aggregation, and contributes to neurodegeneration [61]. Copper binding to cellular proteins, especially membrane and cytoskeletal proteins, disturbs their folding and causes not only subtle structural and mechanical changes to the cell surface and membrane topography but also alters the architecture of the cytoskeleton and cell membranes. Therefore, the nanomechanical measurements of cell elasticity can provide deeper insights into the effects of copper ions on the nanomechanical properties of the entire cell and can serve as valuable biological markers of neurodegenerative changes in targeted cells.

## 5. Conclusions

This study is the first to investigate the effects of flavonol myricitrin on neuroblastoma SH-SY5Y cells exposed to excess copper ions, uncovering both the cellular and molecular mechanisms of its action. Additionally, we employed atomic force microscopy to assess the morphological and nanomechanical properties of SH-SY5Y cells, enabling us to observe neuronal damage at the nanoscale. Our findings highlight the neurotoxic effects of myricitrin and suggest that its pro-oxidative activity plays a central role in cell death.

We demonstrated that flavonol myricitrin exacerbates the toxic effects of copper, leading to decreased cell survival and increased ROS production. The pro-oxidative effects of myricitrin were associated with intracellular calcium overload, resulting in caspase-3/7-dependent apoptosis, increased non-transcriptional p53 activity, and the activation of the p38 MAPK signalling pathway. However, the precise mechanisms require further investigation. Flavonoids, known for their antioxidant properties, are among the most studied natural compounds as potential therapeutics for preventing or alleviating various diseases, including neurodegenerative disorders. However, in the presence of redox active transition metal ions, which are elevated in several neurological and mental illnesses, flavonoids can exhibit prooxidant effects. Thus, further research is essential to better understand their mechanisms of action in the presence of metal ions, which is crucial for achieving safe and effective therapeutic strategies for CNS diseases. Furthermore, it is important to acknowledge the limitations of in vitro models, which may not accurately reflect in vivo conditions. In vivo systems encompass a broader array of protective and regulatory mechanisms that could influence the observed effects. Therefore, future studies are warranted to validate these mechanisms in animal models to better understand their physiological relevance and therapeutic potential.

## Figures and Tables

**Table 1:** Morphological and nanomechanical characteristics of neuroblastoma SH-SY5Y cells.

	Treatment			
	Control Cells	Myricitrin (10 μg/mL)	Cu^2+^ (0.5 mM CuSO_4_)	Myricitrin (10 μg/mL) + Cu^2+^ (0.5 mM CuSO_4_)
*Z*_range_ (*N*_cell_ = 8)/nm	103 ± 24	116 ± 40	37 ± 29	185 ± 50
*R*_q_ (*N*_cell_ = 8)/nm	19.7 ± 1.3	16.8 ± 5.3	4.7 ± 4.8	24.3 ± 6.5
*R*_a_ (*N*_cell_ = 8)/nm	16.7 ± 1.7	13.4 ± 4.3	3.5 ± 3.6	19.0 ± 5.3
*E (N*_cell_ = 7–12)/kPa	4.21 ± 0.17	4.81 ± 0.06	3.4 ± 0.2	3.29 ± 0.08

## Data Availability

The data presented in this study are available in this article.

## Supplementary Materials

The following supporting information can be downloaded at: https://www.mdpi.com/article/10.3390/antiox14010046/s1, Figure S1: The structural formula of myricitrin (C21H20O12).
