# Supplementary figures and images for: Neurotoxic Effect of Myricitrin in Copper-Induced Oxidative Stress Is Mediated by Increased Intracellular Ca2+ Levels and ROS/p53/p38 Axis
Source: Antioxidants (Basel). 2025 Jan 3;14(1):46. doi: 10.3390/antiox14010046 (PMC11763042; doi:10.3390/antiox14010046)

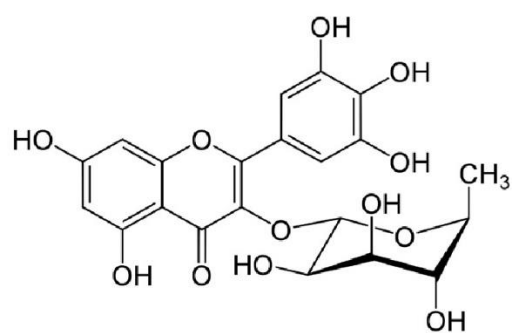

**Supplementary Figure 1.** The structural formula of myricitrin (C<sub>21</sub>H<sub>20</sub>O<sub>12</sub>)

Supplement: Supplementary file 1 [file antioxidants-14-00046-s001.zip › antioxidants-3377453-supplementary.pdf]
